# Significant improvement with ivarmacitinib after suboptimal response to tofacitinib in severe alopecia areata: a case report and literature review

**DOI:** 10.3389/fimmu.2026.1766847

**Published:** 2026-02-17

**Authors:** Zulin Wan, Ying Wang, Dingquan Yang

**Affiliations:** 1 Graduate School, Beijing University of Chinese Medicine, Beijing, China; 2 Department of Dermatology, China-Japan Friendship Hospital, Beijing, China

**Keywords:** alopecia areata, case report, ivarmacitinib, JAK1 selectivity, tofacitinib

## Abstract

Alopecia areata (AA) is a chronic, immune-mediated hair loss disorder, in which the JAK-STAT signaling pathway plays an important pathogenic role. Available agents for AA include minoxidil, corticosteroids, immunosuppressants and Janus kinase (JAK) inhibitors, among others. For adults with severe AA, JAK inhibitors have emerged as cornerstone systemic treatments, but the responses to them are variable. Ivarmacitinib is a novel and highly selective JAK1 inhibitor, which has been approved for AA in China recently with little real-world evidence. We present a case of a patient with severe AA who achieved significant improvement with ivarmacitinib after suboptimal response to tofacitinib, and review previous studies on switching therapy between different JAK inhibitors for AA. This case suggests that ivarmacitinib is a viable alternative for tofacitinib-refractory AA, possibly due to its higher JAK1 selectivity. Further studies are required to define ivarmacitinib’s optimal position in AA treatment algorithm, and to elucidate underlying mechanisms behind JAK inhibitors, which is essential for more personalized and targeted therapy for severe AA.

## 1 Introduction

Alopecia areata (AA) is a chronic, inflammatory, immune-mediated disorder characterized by non-scarring hair loss. In severe cases, it can progress to complete scalp hair loss or whole-body hair loss (alopecia universalis, AU), posing a profound therapeutic challenge and significantly impacting patients’ physical and psychological well-being (1). The hair loss in AA results from T-cell-mediated autoimmune attacks on hair follicles, with the JAK-STAT signaling pathway playing an important role in the pathogenesis of AA.

Available treatments for AA include topical or systemic agents like minoxidil, corticosteroids, immunosuppressants and JAK inhibitors, among others (1). For adults with severe AA, JAK inhibitors have revolutionized treatments, shifting the paradigm from broad immunosuppression to targeted therapy, and now serve as first-line therapy. This shift was heralded by the FDA approval of baricitinib, the first JAK inhibitor for AA (2). However, responses are variable, and some patients exhibit suboptimal improvement. Ivarmacitinib is a novel and highly selective JAK1 inhibitor, which has been approved in China for AA in June, 2025. While clinical trials (3) have proved its efficacy and safety, real-world evidence for AA, particularly its use in patients refractory to other JAK inhibitors, remains scarce.

Here, we report a case of severe AA that showed significant clinical improvement after switching to ivarmacitinib following a suboptimal response to the prior treatment with tofacitinib, highlighting its potential as a salvage therapy. Additionally, we review previous studies on switching to another JAK inhibitor for AA when patients experiencing inadequate response to one JAK inhibitor.

## 2 Case presentation

**Figure 1:**
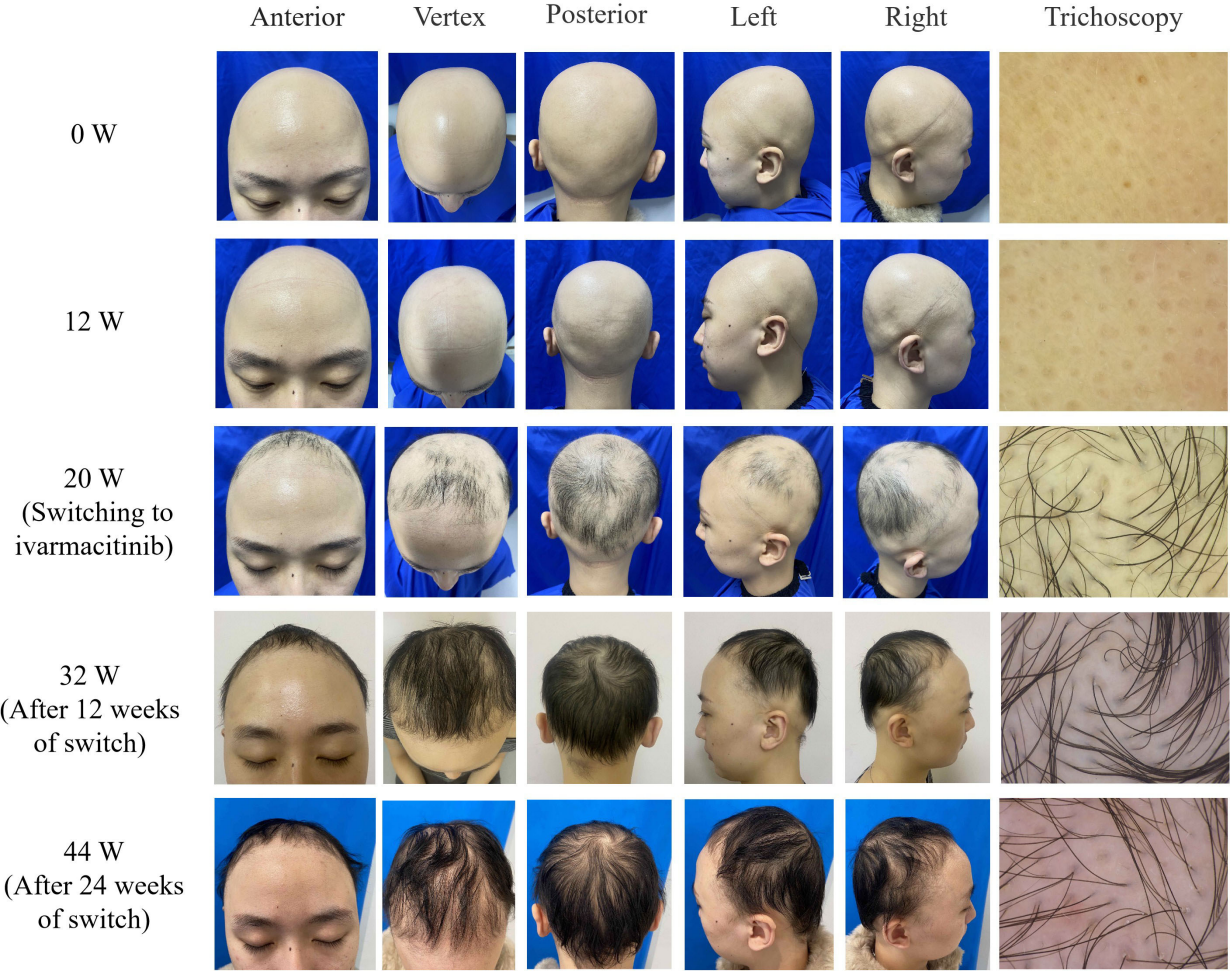
Clinical photographs showing progressive scalp hair regrowth after switching to ivarmacitinib (week 32 and 44) compared to minimal regrowth with tofacitinib (week 12 and 20).

**Figure 2:**
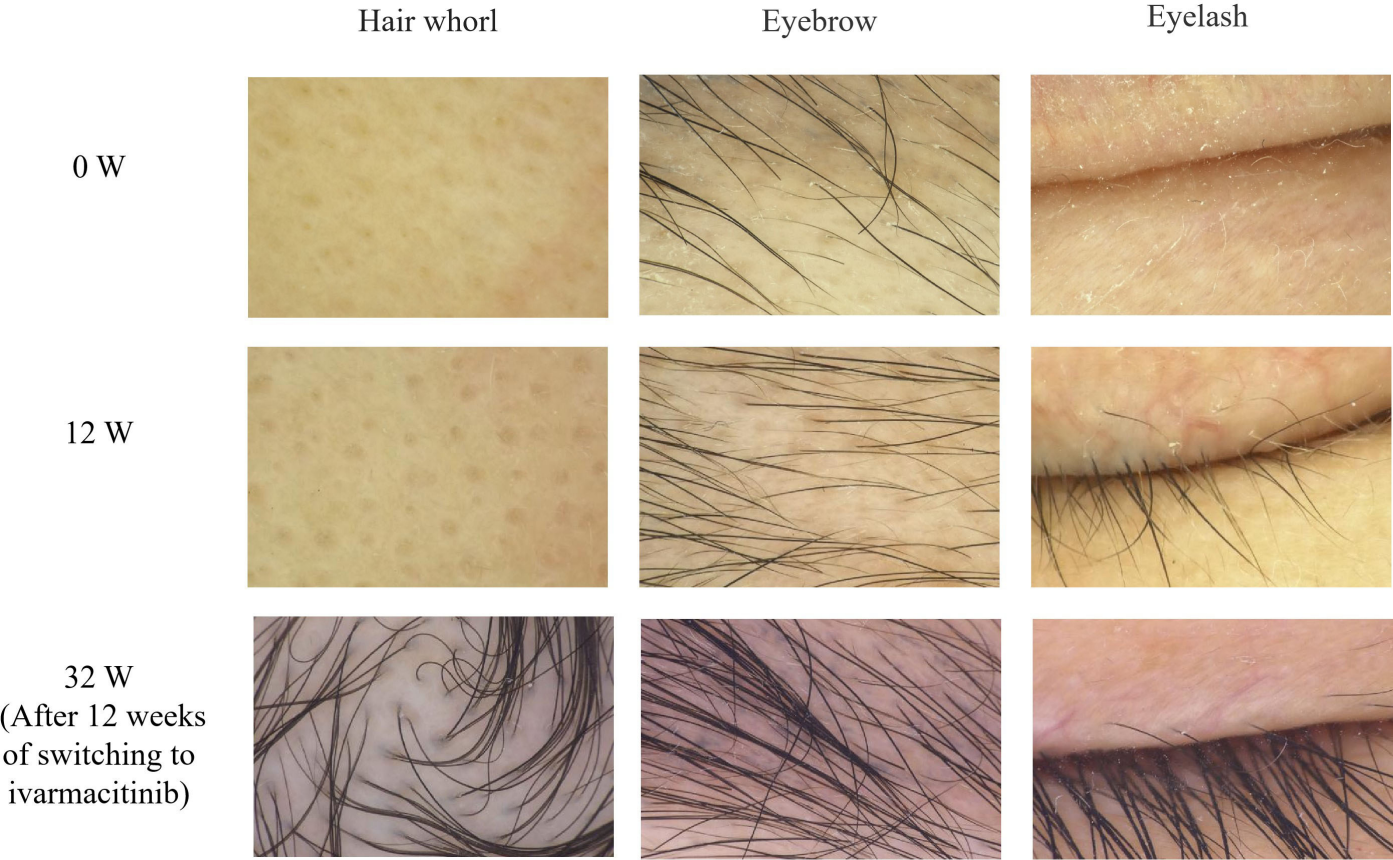
Trichoscopy images of the patient at baseline (week 0), week 12, and week 32 (×40).

A 26-year-old female presented with hair loss over the past 8 years. Initially she experienced patches of hair loss without an apparent cause, which had evolved into diffuse hair loss in half a year due to lack of timely treatment. She visited our clinic for dermatological consultation in January, 2025, and the dermatological examination showed complete scalp hair loss, accompanied by approximately 50% eyebrow and 80% eyelash involvement (**Figure 1**). Her fingernails were pitted and uneven. The Severity of Alopecia Tool (SALT) score was 100. Trichoscopy identified characteristic yellow dots without black dots and broken hairs (**Figure 2**). She was previously healthy, and denied a family history of any hair loss disorder. She had previously used topical minoxidil and hydrocortisone solution, but with no significant effect. The patient was ultimately diagnosed with AU, and was initiated on tofacitinib 5 mg once daily. By week 12, there was no hair regrowth in her scalp, despite partial regrowth of the eyebrows and nearly complete regrowth of both eyelashes (**Figure 1**). The SALT score was still 100, and trichoscopy showed no notable changes from baseline (**Figure 2**). Consequently, the dosage of tofacitinib was increased to 5 mg twice daily. By week 20 (June, 2025), we observed only sparse regrowth on the scalp, with some vellus and nearly no terminal hairs (**Figure 1**). Trichoscopy revealed no obvious changes except some vellus and intermediate-length hairs regrowth. Nevertheless, the SALT score only decreased to 90.

Given the patient’s suboptimal response to tofacitinib and better efficacy of ivarmacitinib in its phase 3 trial than other JAK inhibitors approved for AA in China (3), we decided to change the therapeutic regimen to ivarmacitinib 4 mg once daily after obtaining her informed consent. By week 32 (12 weeks after switch), we observed substantial hair regrowth on her scalp, with a number of intermediate-length and terminal hairs (**Figure 1**). The SALT score decreased to 35, and her fingernails became flatter than before. Trichoscopy revealed more intermediate-length hairs and terminal hairs, with complete regrowth of both eyebrows and both eyelashes (**Figure 2**). The therapeutic regimen was maintained. By week 44 (24 weeks after switch), despite slight hair loss, we observed that the thickness of the hair on the scalp was almost the same as at the previous follow-up (**Figure 1**), and the SALT score was sustained at 35. The improvement of her fingernails was more notable.

Considering systemic corticosteroids are first-line medications for severe alopecia areata, we administered intramuscular compound betamethasone (7 mg each) at week 0, 12 and 20 to improve clinical efficacy and response of the patient. In light of her active hair loss and previous severity, an additional dose was administered concurrently with oral ivarmacitinib at week 32. This adjunctive therapy was intended to provide temporary immunosuppressive support and was not considered the primary therapeutic agent for her long-term management.

Infectious disease screening such as tuberculosis, hepatitis B and C virus, human immunodeficiency virus and syphilis test were performed before tofacitinib therapy. Other laboratory tests including routine blood counts, the function of liver and kidney and lipid panel were performed at each follow-up. All outcomes of laboratory tests mentioned above remained within normal limits or presented negative. Nearly 3 months after initiating tofacitinib and ivarmacitinib respectively, the patient reported mild folliculitis, which was less bothersome during ivarmacitinib treatment. We have prescribed topical mupirocin ointment twice daily to resolve the problem. Apart from this, there was no other adverse events or infections during follow-up.

## 3 Discussion

AA is an autoimmune disorder triggered by a combination of genetic predisposition, immune responses and environmental factors (4). JAK inhibitors have become promising therapies for severe AA, which can inhibit activation of Signal Transducers and Activators of Transcription proteins, thereby blocking the downstream signaling of pro-inflammatory cytokines (5). However, managing severe AA remains challenging due to its refractory nature and high recurrence rate (6).

Ivarmacitinib is a highly selective JAK1 inhibitor that has been approved for atopic dermatitis, rheumatoid arthritis and ankylosing spondylitis (7), and recently received regulatory approval for AA in China. In this case, switching to ivarmacitinib was followed by a marked increase in scalp hair density and a reduction in SALT score of more than 50%, demonstrating its efficacy in severe AA. This suggests that ivarmacitinib is a viable alternative when the response to tofacitinib is inadequate. Furthermore, during ivarmacitinib treatment, the folliculitis that had been reported in clinical trials (3) was less severe compared with the period of tofacitinib therapy, indicating that ivarmacitinib may offer improved tolerability.

The superior response to ivarmacitinib in our patient may be attributed to its high JAK1 selectivity; while tofacitinib, exhibiting inhibitory effect on JAK1, JAK2 and JAK3, is considered as a non-selective JAK inhibitor. It has been proposed that while selective JAK1 and JAK3 inhibitors could potently decrease the frequency of resident memory T cells, which had been noted in AA, JAK2 inhibitors could not achieve the same effect (8). Recent studies have also found that JAK2 can lead to adverse effects due to its ubiquitous expression (9, 10), thus JAK inhibitors with less JAK2 selectivity may be more ideal. With its high selectivity for JAK1, which is 9 times greater than for JAK2, 77 times greater than for JAK3 (7), ivarmacitinib may allow for more potent blockade of key cytokines (e.g. IL-15, IFN-γ) implicated in AA, while minimizing off-target effects on other JAK isoforms (11). Other possible factors, such as the severity of AA, disease duration and genetic predisposition of patients, contribute to inter-individual variability in treatment response. A retrospective study of 13 AA patients showed that while all mild AA patients responded to tofacitinib treatment, it was unsuccessful in half of AU patients (12), suggesting patients with severe AA may not respond well to tofacitinib.

For patients with a suboptimal response to one JAK inhibitor, switching to an alternative JAK inhibitor may represent a practical therapeutic strategy, as supported by several retrospective studies and case reports. For instance, one report (13) described a 50-year-old woman with severe AA who, after failing to respond to tofacitinib and dupilumab, achieved complete regrowth of scalp hair, eyebrows, and eyelashes following a switch to ruxolitinib. Another recent case reported by Yao et al. (14) involved a patient with AU who initially responded to tofacitinib by week 28 (SALT = 5) but relapsed by week 40 (SALT = 30); subsequent switching to ritlecitinib led to a satisfactory response by week 55 (SALT = 5). A retrospective study (15) further indicated that for most patients who switched from tofacitinib to baricitinib or switched from baricitinib to tofacitinib, complete or partial responses were achieved despite some adverse events. Additionally, a case series (16) demonstrated that ruxolitinib was effective in 5 out of 8 patients with severe AA who had previously received tofacitinib (**Table 1**). These findings show that switching to another JAK inhibitor is a promising strategy for AA patients who have failed with one JAK inhibitor. However, the sample sizes of most current studies are small. Studies with larger sample or involving more JAK inhibitors are needed to further explore the efficacy and safety of this strategy.

**Table 1:** Overview of reported cases and studies on switching between different JAK inhibitors for AA.

Study (Author)	No. of patients	Age (years)	Duration of AA (years)	Prior JAK inhibitor (Response)	Switched to JAK inhibitor	Outcome after switch	Adverse events
Liu LY, et al. (16)	8 (4M/4F)	14-57	0.5-5	Tofacitinib (6 pts; 3 substantial regrowth) or none (2 pts)	Ruxolitinib	5 (near-)complete regrowth; 3 no regrowth	Upper respiratory infections, weight gain, acne, easy bruising and fatigue
Peterson D, et al. (13)	1 (F)	50	30+	Tofacitinib + Dupilumab (No response)	Ruxolitinib	Complete regrowth (SALT 100→0)	Not mentioned
Kazmi A, et al. (15)	77 (48M/29F)	39.3 (mean)	12.9 (mean)	Tofacitinib (73 pts; 30 complete response, 35 partial response, 8 no response) or Baricitinib (4 pts; 1 partial response, 3 no response)	Baricitinib or Tofacitinib	Majority achieved complete/partial response	Hypercholesterolemia, transaminitis, neutropenia, headache, lymphopenia and acne
Yao L, et al. (14)	1 (F)	26	9	Tofacitinib (Relapsed after initial response)	Ritlecitinib	SALT decreased from 10 to 5	None
Our Case	1 (F)	26	8	Tofacitinib (Suboptimal response: SALT 100→90)	Ivarmacitinib	Substantial regrowth (SALT 90→35)	Mild scalp folliculitis

M, Male; F, Female; pts, patients; SALT, The Severity of Alopecia Tool.

To the best of our knowledge, this is the first real-world evidence of switching treatment with ivarmacitinib for severe AA. Since this is merely a single case report, we acknowledge that the level of evidence is low. Concurrent betamethasone injections are a confounding factor, and delayed onset of tofacitinib effect must be considered. Because potent corticosteroids usually have a rapid onset of action, we would have observed substantial hair regrowth on the scalp at week 12 or week 20 if betamethasone injection had taken effect. However, there was little hair regrowth by week 20, so it is less likely that betamethasone would play a major role. In contrast, adequate response exhibited after switching to ivarmacitinib and therefore the improvement is more attributable to it. Meanwhile, although it is established that JAK inhibitors exhibit a delayed onset of action, with significant regrowth typically observed between 3 to 6 months of treatment as demonstrated in pivotal trials for baricitinib and ritlecitinib (17, 18), our assessment of a suboptimal response to tofacitinib was made after the 20-week (approximately 5-month) follow-up, already close to the maximum duration of its delayed onset. The significant improvement observed after 12 weeks of ivarmacitinib treatment makes it less likely that tofacitinib played the primary role. Further head-to-head comparative studies, larger and longer real-world cohorts are required to confirm whether the efficacy and safety of ivarmacitinib are better than those of tofacitinib, even if AA patients subsequently discontinue receiving ivarmacitinib treatment.

## 4 Conclusion

This case report provides real-world evidence that ivarmacitinib can induce significant hair regrowth in patients with severe, tofacitinib-refractory AA, suggesting that in patients with suboptimal response to one JAK inhibitor, switching to another agent, such as the highly JAK-selective inhibitor, represents a promising therapeutic strategy. Ivarmacitinib’s high JAK1 selectivity may offer a favorable efficacy and tolerability profile. Further studies are warranted to validate these findings and to elucidate underlying mechanisms behind different JAK inhibitors, which is essential for more personalized and targeted therapies to achieve better therapeutic effects.

## Data Availability

The raw data supporting the conclusions of this article will be made available by the authors, without undue reservation.

## References

[1] Zhou C Li X Wang C Zhang J . Alopecia areata: an update on etiopathogenesis, diagnosis, and management. Clin Rev Allergy Immunol. (2021) 61:403–23. doi: 10.1007/s12016-021-08883-0, PMID: 34403083

[2] Gencebay G Bahalı AG Dizman D Özay M Su Küçük Ö . Baricitinib for the treatment of moderate-to-severe alopecia areata. Balkan Med J. (2025) 42:176–9. doi: 10.4274/balkanmedj.galenos.2025.2024-9-31, PMID: 39810568 PMC11881537

[3] Zhou C Yang C Fan W Wu J Yang D Jin H . Ivarmacitinib for the treatment of adults with severe alopecia areata: Results from a phase 3 trial. J Am Acad Dermatol. (2026) 94:161–71. doi: 10.1016/j.jaad.2025.09.044, PMID: 40976531

[4] Ma T Zhang T Miao F Liu J Zhu Q Chen Z . Alopecia areata: pathogenesis, diagnosis, and therapies. MedComm. (2025) 6:e70182. doi: 10.1002/mco2.70182, PMID: 40260013 PMC12010142

[5] Cai L Wei Y Zhao M Zhuo J Tao X Lin M . Case report: Dupilumab therapy for alopecia areata in a 4-year-old patient resistant to baricitinib. Front Med. (2023) 10:1253795. doi: 10.3389/fmed.2023.1253795, PMID: 37877023 PMC10591075

[6] Huang J Tan Z Tang Y Shi W . Screening for latent infectious disease in patients with alopecia areata before initiating JAK inhibitors therapy: a single-center real-world retrospective study. Front Med. (2023) 10:1287139. doi: 10.3389/fmed.2023.1287139, PMID: 37920596 PMC10619649

[7] Keam SJ . Ivarmacitinib sulfate: first approval. Drugs. (2025) 85:1163–70. doi: 10.1007/s40265-025-02202-z, PMID: 40542222

[8] Sardana K Bathula S Khurana A . Which is the ideal JAK inhibitor for alopecia areata - baricitinib, tofacitinib, ritlecitinib or ifidancitinib - revisiting the immunomechanisms of the JAK pathway. Indian Dermatol Online J. (2023) 14:465–74. doi: 10.4103/idoj.idoj_452_22, PMID: 37521227 PMC10373824

[9] Perrone M Sergio S Pranzo B Tarantino A Loglisci G Matera R . JAK2 46/1 (GGCC) haplotype in oncogenesis, as risk stratifier, and indicator for drug resistance in myeloproliferative neoplasms. Int J Mol Sci. (2025) 26:10337. doi: 10.3390/ijms262110337, PMID: 41226376 PMC12609092

[10] Zimolova V Burocziova M Berkova L Grusanovic S Gursky J Janotka L . Germline Jak2-R1063H mutation interferes with normal hematopoietic development and increases risk of thrombosis and leukemic transformation. Leukemia. (2025) 39:2745–57. doi: 10.1038/s41375-025-02737-w, PMID: 40841769 PMC12589134

[11] Dai Z Chen J Chang Y Christiano AM . Selective inhibition of JAK3 signaling is sufficient to reverse alopecia areata. JCI Insight. (2021) 6:e142205. doi: 10.1172/jci.insight.142205, PMID: 33830087 PMC8119218

[12] Dincer Rota D Emeksiz MAC Erdogan FG Yildirim D . Experience with oral tofacitinib in severe alopecia areata with different clinical responses. J Cosmet Dermatol. (2021) 20:3026–33. doi: 10.1111/jocd.13966, PMID: 33533091

[13] Peterson D Powell M King B . Less is more? Failure of one JAK inhibitor does not predict failure of another one in a patient with alopecia areata. Dermatol Ther. (2021) 34:e15062. doi: 10.1111/dth.15062, PMID: 34250709

[14] Yao L He J Lan N Lv Y . Considerations for the treatment strategy of relapse after tofacitinib therapy in alopecia areata. J Cosmet Dermatol. (2025) 24:e70234. doi: 10.1111/jocd.70234, PMID: 40599045 PMC12215230

[15] Kazmi A Moussa A Bokhari L Bhoyrul B Joseph S Chitreddy V . Switching between tofacitinib and baricitinib in alopecia areata: A review of clinical response. J Am Acad Dermatol. (2023) 89:1248–50. doi: 10.1016/j.jaad.2023.03.041, PMID: 37024053

[16] Liu LY King BA . Ruxolitinib for the treatment of severe alopecia areata. J Am Acad Dermatol. (2019) 80:566–8. doi: 10.1016/j.jaad.2018.08.040, PMID: 30195572

[17] Kwon O Senna MM Sinclair R Ito T Dutronc Y Lin CY . Efficacy and safety of baricitinib in patients with severe alopecia areata over 52 weeks of continuous therapy in two phase III trials (BRAVE-AA1 and BRAVE-AA2). Am J Clin Dermatol. (2023) 24:443–51. doi: 10.1007/s40257-023-00764-w, PMID: 36855020 PMC9974384

[18] Hordinsky M Hebert AA Gooderham M Kwon O Murashkin N Fang H . Efficacy and safety of ritlecitinib in adolescents with alopecia areata: Results from the ALLEGRO phase 2b/3 randomized, double-blind, placebo-controlled trial. Pediatr Dermatol. (2023) 40:1003–9. doi: 10.1111/pde.15378, PMID: 37455588

